# Synthetic Slag Production Method Based on a Solid Waste Mix Vitrification for the Manufacturing of Slag-Cement

**DOI:** 10.3390/ma12020208

**Published:** 2019-01-09

**Authors:** Mónica Rodríguez-Galán, Bernabé Alonso-Fariñas, Francisco M. Baena-Moreno, Carlos Leiva, Benito Navarrete, Luis F. Vilches

**Affiliations:** Chemical and Environmental Engineering Department, Technical School of Engineering, University of Seville, C/Camino de los Descubrimientos s/n, 41092 Sevilla, Spain; bernabeaf@us.es (B.A.-F.); fbaena2@us.es (F.M.B.-M.); cleiva@us.es (C.L.); bnavarrete@us.es (B.N.); luisvilches@us.es (L.F.V.)

**Keywords:** blast furnace slag, slag-cement, synthetic slags, waste recycling

## Abstract

Herein an innovative process to develop a potential vitreous material with cementing properties is proposed. This process paves a production path through melting industrial waste and subsequently cooling the casting in water. The idea erases the need to reduce the environmental impact of the cement industry in terms of natural resources consumption as well as the re-utilization of abandoned wastes from other industries. The recycled industrial wastes were selected according to the amount of waste produced in the industrial field and its suitable chemical composition, such as construction and demolition waste and/or shells from shellfish. As a main result, the mechanical properties showed by our novel material were worse than those reported by blast furnace slag (25–28 MPa for two different proportions) for seven days and better (43–52 MPa for two different proportions) for 28 days. The rest of the properties evaluated were in agreement with the standards’ requirements. Hence, this novel process would help to minimize the environmental impact of these wastes at the same time that their use in the cement industry would reduce the consumption of raw materials.

## 1. Introduction

The cement industry has the challenge of reducing the environmental impact of its activity, both at the levels of intensive energy consumption and greenhouse gas (GHG) emissions reduction, as well as in the reduction of the use of raw materials from natural sources. This has motivated, for example, the development of non-Portland cement which is prepared using powders that are normally used as supplementary cementitious materials [1]. Cement substitution by industrial by-products or wastes has become a critical issue under a circular economy approach [2]. Some of the cement substitutes employed or under study are combustion ash [3], silica fume [4], glass waste [5], spent fluid catalytic cracking (FCC) catalyst [6] and blast furnace slag (BFS) [7].

Blast furnace slag is one of the most abundant solid by-products of the iron-making process, with between 300 and 350 kg of BFS per ton of pig iron produced [8]. Blast furnace slag from the iron making industry is mainly composed of CaO, SiO_2_, and Al_2_O_3_ [9]. Due to its latent hydraulic activity, it is considered an excellent substitute for Portland cement [10]. Furthermore, BFS can be employed to produce high-performance concrete (HPC) with better properties than a net-cement product by increasing workability and strength along with by reducing hydration heat, permeability, and porosity [4,10]. Greater durability was found in slag-cement mortar than net-cement mortar. Better strength/heat was reported [11] by adding BFS.

Authors such as Lin et al. [12,13,14] and Lee et al. [15,16,17,18] have proposed the production of a synthetic slag vitrified from wastes. They have successfully studied the performance of cement substitute for synthetic slags obtained by the melting and subsequent cooling and grinding of municipal solid waste incineration (MSWI) fly ash [13,17]. Additionally, they have proposed adjusting the synthetic slag composition, and then optimizing its performance as a cement substitute by melting different combinations of MSWI fly ash with CaO or Al_2_O_3_ [12] or other wastes, such as MSWI scrubber ash [15,16], fly-ash of foundry sand, a by-product of ferrous and non-ferrous metal casting industries which can be used as an alternative to natural sand in concrete [18,19], light emitting diodes (LEDs), and production sludge [20].

The main objective of this research work is to develop a new potential method to obtain a synthetic slag (SS) from a waste mixture vitrified with similar physicochemical properties to that of BFS. Three different wastes have been chosen as raw materials in order to facilitate their recycling as cement substitutes and reduce the amount of waste sent to landfills. These wastes are: construction and demolition waste (CDW) as a main source of silicon (SiO_2_), the solid waste stream generated in an aluminum recovery plant (AW) as the main source of aluminum (Al_2_O_3_), and mussel shell waste from the aquiculture industry (MS) as the main source of calcium (CaO). The CDW comes mainly from demolition of buildings or rejects of materials of new construction or small reforms in buildings. This type of waste is carried in its main part to landfills, causing a negative visual and landscape effect, in addition to the negative environmental impact, which rejects materials that, with a specific treatment, could be recycled. In general, there are two groups of CDW that are used in different applications: recycled aggregates and recycled ceramics or mixed aggregates. In particular, a mixture of fines smaller than 5 mm is used as cement component. This mixture is pulverized to obtain particles of similar size to BFS, fly ash or gypsum, which are other components used in the manufacture of cement [21].

There are studies related to the partial substitution of cement by CDW or ceramic waste. This is the case of Medina et al. [22] in which the replacement of between 10% and 20% of the cement by CDW or by ceramic waste was considered. In this case, the study focused on the rheological and calorimetric behavior of the different mixtures. The behavior of the substitution of one or the other residue offers completely opposite results; while the addition of ceramic residues reduces plasticity and delays hydration reactions, the addition of CDW supposes an increase in the tension of the yield limits and accelerates the hydration reactions of the mixture.

According to this it seems that the field of recycling of the CDW in the manufacture of cements is a field of research with a great potential for its future application. The second waste used (AW) is a high alumina mineral waste generated in the process of recycling salt slags from aluminum melting (secondary aluminum). This waste has a high content of alumina and it can be used in a lot of high value applications. The variability in the chemical composition depends on the raw material used [23].

In the case of waste derived from aquiculture industry, mussel shells are used in construction, such as road surfaces [24] or as fine aggregate in the production of cement mortars [25,26]. Another application of this type of waste is its use in the manufacture of construction elements resistant to fire, mixing constituents from mollusk shells with binders and with water absorbing additives [27]. Another study like the one developed by Li et al. [28] presents the possibility of using ash from oyster shells as a substitute for lime in the manufacture of fly ash bricks. Therefore, the use of mollusk shells in the construction sector has a lot of potential fields of study.

The obtained materials were compared in terms of specific surface, particle size distribution, chemical composition, and vitreous phase percentage with a real BFS which come from the ArcelorMittal factory in Avilés (Asturias) and were provide by cement Portland Valderrivas (Alcalá de Guadaira, Seville, Spain). The performance of the synthetic slag in a slag-cement mortar with 25% and 50% replacement was evaluated according to the European standard EN 197-4: 2005 [29] and using both net-cement mortar and a slag-cement mortar made with a real BFS as reference. On the basis of this work, potential future industrial applications can be developed as the environmental impact of using these wastes would be quite positive.

## 2. Materials and Methods

As previously stated, a mixture of three different wastes were used in order to manufacture a material with similar properties to the BFS.

Construction and demolition waste were used as a source of silicon. A typical mixed aggregate sample was taken from the Environmental Complex of the Mancomunidad de la Vega, which is located between Guillena and Burguillos (Seville, Spain). Construction and demolition waste were taken from the first screening in the recycling plant, where plastics and wood were separated from the rest of the material, so the CDW used contained aggregates of concrete, mortars, ceramics, and gypsum. The AW used in the study came from a processing plant and was obtained from a recycling process of salt slags in order to recover free metals and melting slags. Mussel shell waste was supplied by a seashell recycling company. The shells were subjected to a heat treatment of 150 °C for sterilization prior to use, and they were grinded in order to get particles between 0.1 and 0.5 mm in size.

In addition to the three mentioned wastes, commercial Portland cement type I 42.5 MPa (C), nominal strength according to the EN 197-1:2011 [30], and standardized sand were used for preparing the mortars used in the evaluation of mechanical properties.

For the production of cementitious material, a manufacturing system that simulates the conditions for the production of BFS in the industry were developed. First, heating was performed to temperatures above the melting point of the waste mixture in an electric furnace (ATERCAN S.C., Seville, Spain). Then, the molten material was subjected to a rapid cooling which gave it a high vitreous phase. A high percentage in the vitreous phase provided high reactivity to the material to be used as an addition to cement. Once the cementitious material was obtained, it was used as an addition to the cement in different proportions (25% and 50%) and the physical, chemical, and mechanical properties were evaluated based on standards.

### 2.1. Waste Characterization: Chemical Composition and Thermal Properties

The chemical composition of the wastes was determined using an X-ray Fluorescence Spectrometer (XRF, Model AXIOS, Panalytical, Lelyweg, Almelo, The Netherlands). Thermal analyses were performed in order to study the behavior of the wastes during the process of heating and melting in order to establish the thermokinetic conditions of melting and to identify the occurrence of possible physical and chemical changes, both in the solid phase and molten phase throughout this process. For this purpose, a thermo-gravimetric study (TG-SDTA Q600 TA Instruments, New Castle, DE, USA) of each waste and mixture was carried out with a heating rate of 20 °C/min while using air as a purging gas. In order to determine the melting point of the mixture of waste, a heating microscopy was carried out in a furnace (ATERCAN S.C., Seville, Spain) with silicon carbide resistances and 1700 °C as maximum operational temperature with a video-camera system (model Canon MD130 Mini DV, Tokyo, Japan) coupled to the furnace. An image analysis program was used to study the evolution of the fusion of solid samples as a function of temperature. Thus, different material characteristics were obtained, such as deformation temperature, the formation temperature sphere or hemisphere, and the flow temperature or sintering starting.

### 2.2. Waste Mixture Adjustment for Synthetic Slag (SS) Manufacturing

**Figure 1:**
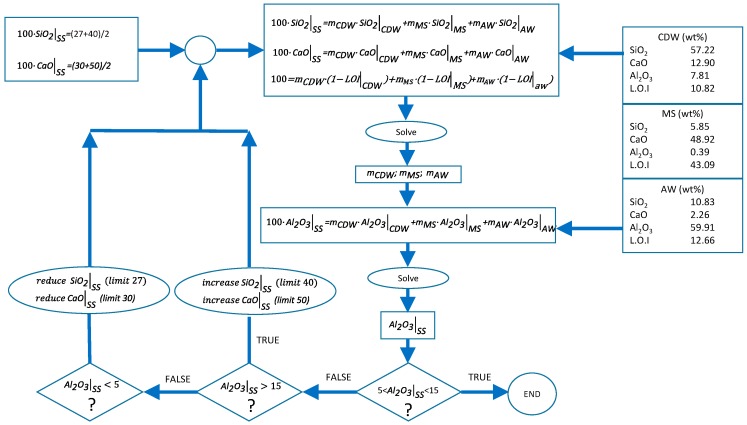
Methodology for waste mixture adjustment.

A waste mixture adjustment methodology was defined in order to obtain an SS with similar chemical composition to BFSs. This methodology is schematized in Figure 1. Typical weight percent composition ranges for BFS are shown in Table 1 [31,32,33,34,35].

As shown in Figure 1, the SiO_2_, CaO, Al_2_O_3_, and loss on ignition (L.O.I) percentages of each of the wastes were employed as inputs for solving the mass balances. The material lost during the melting process was supposed to be equal to the sum of the loss of ignition values of each of the wastes. The calculation methodology consists of an iterative process. Firstly, SiO_2_, CaO, and global mass balances were calculated by imposing initial values for both SiO_2_ and CaO percentages in SS. These initial values were the intermedium concentration of the ranges reported in Table 1. Secondly, the obtained values of m_CDW_ (wt % CDW), m_MS_ (wt % MS), and m_AW_ (wt % AW) were employed to check if the Al_2_O_3_ concentration was in the range shown in Table 1. If this concentration was in the range, then the calculation was finished. Otherwise, the initial values of SiO_2_ and CaO were increased or reduced if the Al_2_O_3_ was too high or too low, respectively. Following this methodology, the following waste mixture (WM) adjustment was employed to produce the synthetic slag: 37 wt % CDW, 53 wt % of MS, and AW 10 wt %.

### 2.3. Melting and Cooling of the Waste Mixture

**Figure 2:**
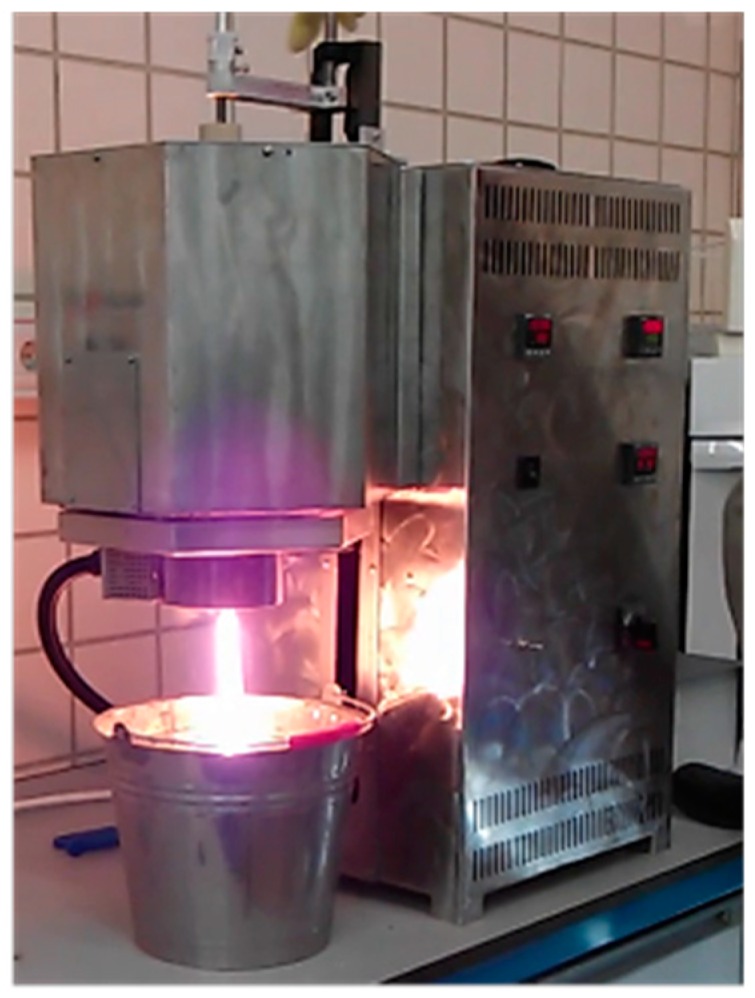
Furnace and cooling system.

The furnace used for the melting of wastes was designed and manufactured specifically for this research and consists of six electrical resistances located in the upper part of the equipment and arranged in a hexagonal shape on the sides of the fusion chamber. The melting pot used for the fusion was made of silicon carbide and was placed on a vertically opening platform. Table 2 summarizes the most important characteristics of the furnace. The discharge of the material, once melted, was done through the bottom of the oven. After the fusion of the material, a water cooling was carried out in order to cool the material as fast as possible to confer the highest proportion to the vitreous phase. This cooling was performed by dropping the casting molten material directly into a container full of water. The relationship between the mass of water and the molten material was about 10:1. Figure 2 shows the furnace and the cooling system used to manufacture the SS.

### 2.4. Characterization of the SS: Specific Surface, Particle Size Distribution, Chemical Composition, and Vitreous Phase

After fusion and cooling with water, the material was carried to an oven for drying at 105 °C for 24 h. Then the material was milled to adjust the particle size to the requirements of the regulations. The first milling step obtained a material smaller than 3 mm. In a second operation, a vibratory mill (EQUILAB Madrid, Spain) was used, and programmed with a speed of 1600 rpm for 3 min. The final sizing is done in a third step in a ball mill (RMU, Bergamo, Italy). The minimum size required for the material was imposed below 32 microns. The determination of the specific surface area was made based on the air permeability method (Blaine method), according to EN 196-6:2010 [36]. The research employed specific surface material similar to cement, above 2750 mg/cm^2^, which is the minimum established by the standard additions of blast furnace slag-cements.

Particle size distribution, chemical composition, and characterization of vitreous phase were carried out at the Technology and Innovation Research Center of the University of Seville. The particle size distribution was analyzed by a Mastersizer 2000 particle size analyzer (Norcross, GA, USA). This equipment allows particle size analysis by the laser diffraction technique and can measure particles with sizes between 0.02 and 2000 microns. Chemical composition was determined using an X-ray fluorescence spectrometer (XRF, Model AXIOS, Panalytical). For the chemical characterization of the waste mixture, a technique of X-ray fluorescence was used with a sequential spectrophotometer. The characterization of the proportion of vitreous phase was estimated using X-ray diffraction. The experimental device used for the determination was a D8 Advance A25 (Bruker Company, Billerica, MA, USA). For this calculation a semi-quantitative method was employed to determine the area under the curve taken as a basic of the diffractogram.

### 2.5. Evaluation of SS for Use as an Addition to the Cement: Evaluation of Mechanical, Physical, and Chemical Properties

Different mixture proportions of SS and C were evaluated to test mechanical, physical, and chemical properties according to the relevant regulations. The tested samples were compared to mixtures with the same proportions of blast furnace slags and cement. All the samples were manufactured using a water/solid ratio of 50%. The mixture proportions are listed in Table 3.

### 2.6. Mechanical Properties

The mechanical properties in the form of unconfined compressive strength (UCS) of the mixture between C and the SS made from waste were evaluated according to EN 196-1:2005 [37], using a compression test machine (Suzpecar, MEM-102/50 t, Madrid, Spain) to determine the UCS with prismatic specimens (40 mm × 40 mm × 160 mm). The mechanical compression requirements defined in Reference [29], makes a distinction between nominal resistance and initial resistance. In the first case, the nominal resistance is defined as the UCS, determined according to Reference [37] at 28 days. In the case of the initial resistance, it is defined as the UCS at 2 and 7 days, with the method used for determining, in both cases, according to Reference [37]. In this study, the UCS was determined on 7- and 28-day-old samples; the UCS after 2 days was not tested because the mortar did not have enough consistency.

### 2.7. Physical Properties

Three physical properties were evaluated according to Reference [29]. The initial setting time and the volume stability were determined following the procedure described in the European standard EN 196-3:2005 + A1:2008 [38], using a Vicat system to measure the initial setting time and the Le Chatelier equipment (Sistemas de Ensayo, Madrid, Spain), described in this standard, to know if the material changes its volume. The heat of hydration was determined according to EN 196-8: 2003 at 7 days [39].

### 2.8. Chemical Properties

The chemical properties of the material were determined according to Reference [29] and compared to the limit values for loss of ignition (established at ≤5.0%), insoluble residue (≤5.0%), sulfate ion content (as SO_3_) (≤4.0%), and content in (≤0.10%) chloride ion. The methodology for determining the loss of ignition, insoluble residue, and sulfate ion content is collected in EN 196-2:2006 [40] while the chloride ion was determined by a potentiometric method according to UNE 80213:2010 [41].

### 2.9. Emission of Dangerous Substances (Heavy Metals) and Emission of Radioactivity

Although in the normative use of cement the necessity to realize environmental tests is not mentioned, these were considered due to the nature of the used materials. The Netherlands Leaching Test (NEN 7345) [42] was carried out in order to prove that the effluents extracted by water action should be contamination-free, and therefore guarantee its use in the building industry. Another reference environmental test was a radioactive test, in which the radiation of the materials used was determined. The evaluation of the compliance of a specific building material with the limits of international recommendations was carried out using the activity concentration index “I”. This index is expressed in terms of activity concentrations of the three major natural radionuclides: Ra-226, Th-232, and K-40, according to the Equation (1):

(1) $$I=\frac{C_{Ra-226}}{300}+\frac{C_{Th-232}}{200}+\frac{C_{K-40}}{3000}$$

where C_Ra-226_, C_Th-232_, and C_K-40_ were the activity concentrations of Ra-226, Th-232, and K-40 in Bq·kg^−1^ in the materials tested [43].

## 3. Results and Discussion

### 3.1. Waste Characterization: Chemical Composition and Thermal Properties

The chemical composition of wastes used is collected in Table 4, which shows the major content of SiO_2_, CaO, and Al_2_O_3_ of CDW, AW, and MS, respectively.

**Figure 3:**
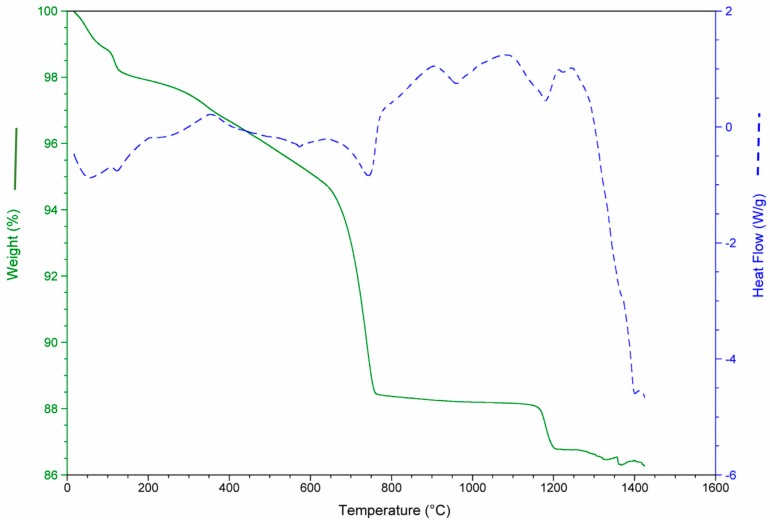
Mass loss and heat flow of construction and demolition waste (CDW).

**Figure 4:**
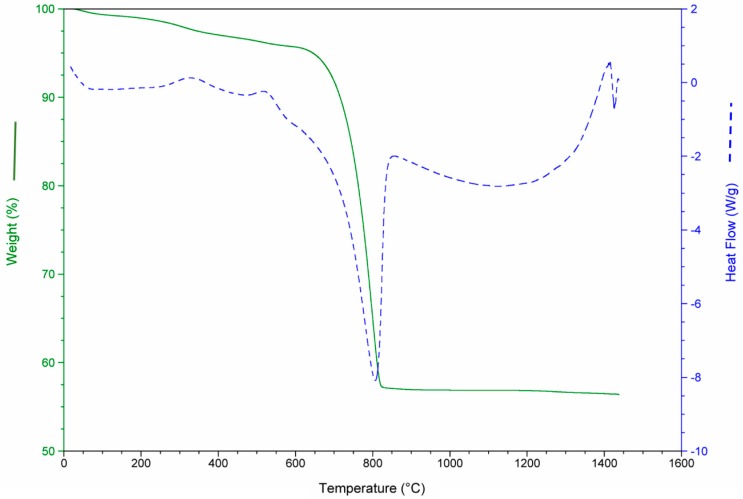
Mass loss and heat flow of aquiculture industry (MS).

**Figure 5:**
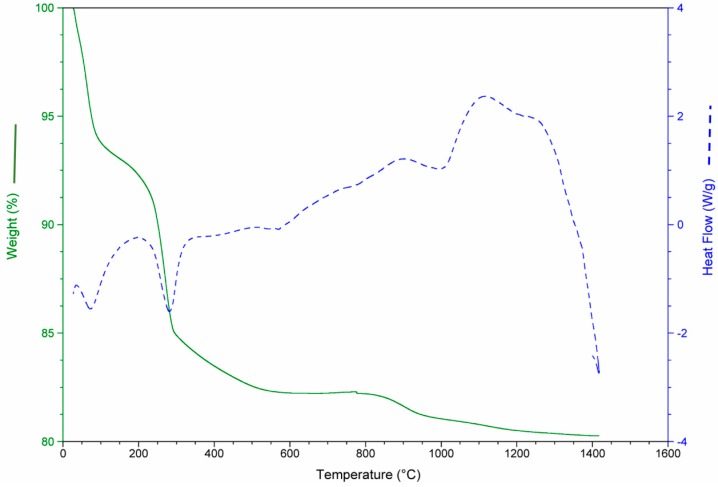
Mass loss and heat flow of aluminum recovery plant (AW).

**Figure 6:**
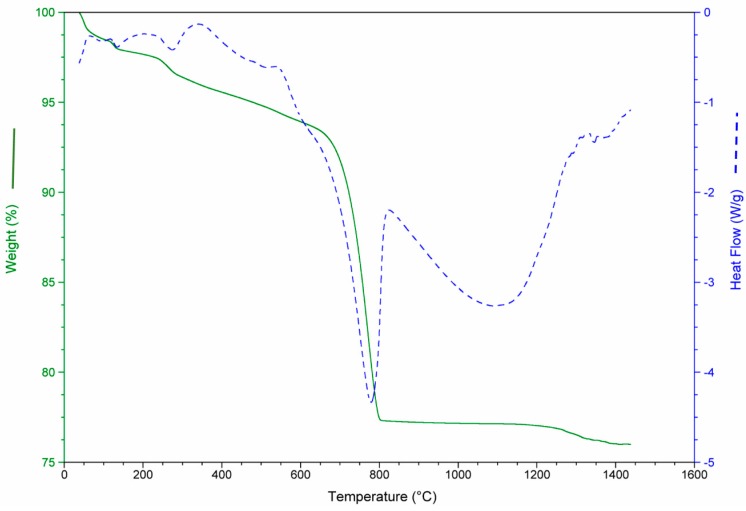
Mass loss and heat flow of the waste mixture (WM).

The results of the thermo-gravimetric studies of each waste and of the waste mixtures are presented in Figure 3, Figure 4, Figure 5 and Figure 6 in order to analyze the physical and chemical transformations in the process of heating through fusion.

Figure 3 shows mass loss and heat flow of the CDW. At the beginning (0–200 °C), the sample weight decreased due to the water loss (moisture and chemically bound water mainly in the form of CaSO_4_·2H_2_O). Between 600 and 800 °C, the decomposition of the CaCO_3_ (calcite) produced a substantial mass loss. The main mass loss was due to the de-carbonation of the calcite (14%).

The MS consists almost entirely of carbonate (aragonite and calcite). For this reason, the de-carbonation produced the most significant mass loss in this waste, as shown in Figure 4, around 43%. It should be noted in all cases that the values of mass loss obtained for each waste were consistent with the calcination losses shown in Table 4.

Figure 5 demonstrates that the mass loss in AW occurred from the beginning of heating. This was due to the transformation of aluminum hydroxides in oxides and water. The total mass loss of AW was around 18%. The peaks of heat flow are associated with loss in moisture which results in the polymorphic transformation of quartz and de-carbonation of calcite.

In addition, a thermo-gravimetric study was performed on a sample with the mixture of the three wastes (SS). The results are shown in Figure 6, where the additive character of the transformations of each one of the wastes previously exposed can be observed.

In order to determine the melting temperature of the waste mixture (WM), a study of microscopy heating was performed to gather data on the melting temperature as well as the temperature ranges in which the viscosity was suitable to allow the melted material to flow.

**Figure 7:**
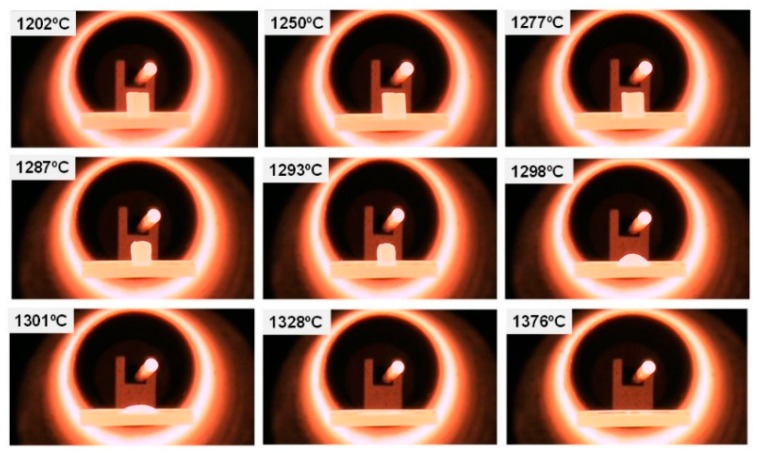
Heating microscopy.

The fusion of the WM with a particle size below 32 microns, to improve the homogeneity of the mixture, was carried out in an electric furnace, and a silicon carbide melting pot was used to contain the material. The furnace reached maximum temperatures of 1650 °C with a lower opening for the exit of the molten material. The heating microscopy tests determined the melting temperature of the material at around 1300 °C, as can be seen in the frames shown in Figure 7. The temperature adopted for melting the material was 1500 °C in order to ensure its correct creeping through the silicon carbide.

### 3.2. Synthetic Slag Characterization: Specific Surface, Particle Size Distribution, Chemical Composition, and Vitreous Phase

The specific surface of the material was an important property which affected the behavior of the mechanical properties of the resulting materials. The comparison between the UCS of SS and BFS mortars was conducted with samples which contained a specific surface larger than 4000 mg/cm^2^, this specific surface value was used because it was obtained after milling process, which was above the minimum established by the standards.

The specific surface is a very important parameter in the evolution of the compressive strength in cement pastes. The hydration process begins at the surface of the particles, so that the hydration rate will increase as the surface of contact between the particles and water increases, contributing mainly to the development of UCS [44].

**Figure 8:**
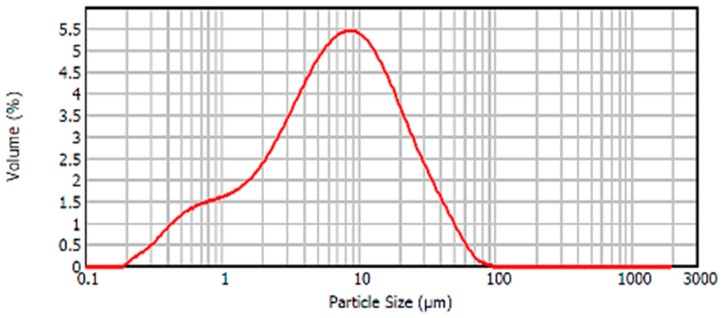
Particle size distribution BFS.

In relation to the particle size distribution of the material, the particle size distribution curve of the BFS is shown in Figure 8. It was observed that this curve presented a Gaussian distribution with a peak in the curve, corresponding to the maximum volume of particles, associated with a size corresponding to 8–9 microns. Most of the particles were distributed in sizes between 0.5 and 40 microns.

**Figure 9:**
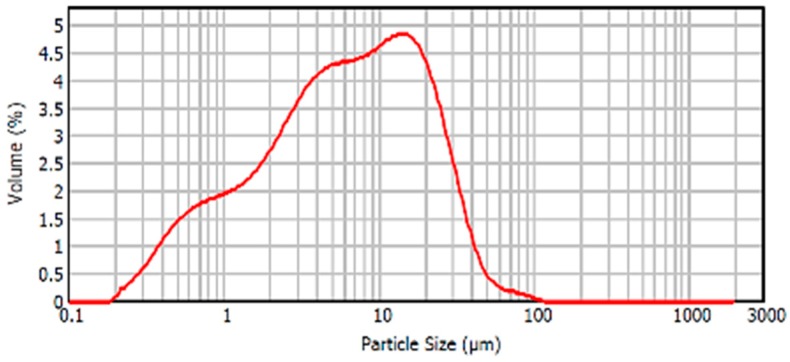
Particle size distribution SS.

Figure 9 shows the particle size distribution of SS. The peak of the curve was 15 microns and did not have particles larger than 100 microns. The high proportion of particles was between 10 and 45 microns and presented a high percentage of particles below 10 microns.

**Figure 10:**
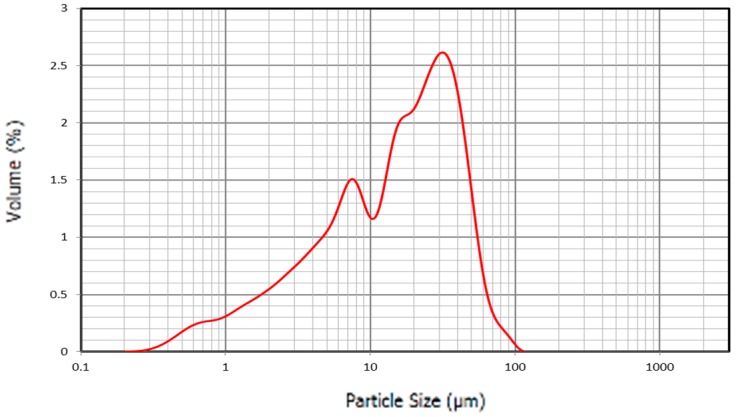
Particle size distribution C.

The particle size distribution of cement used in the study is shown in Figure 10 as additional information.

Table 5 summarizes the basic compositions of the materials used in this study. In both cases of SS and BFS the composition of the three major compounds, SiO_2_, CaO, and Al_2_O_3_ were similar, which allowed for a good comparison of physical, chemical, and mechanical properties in the mixture with cement with the same proportions.

The characterization of the vitreous phase of the SS is necessary to explain the cementing behavior. The vitreous phase confers to the materials the reactive character to be used as additives in cement. The vitreous phase ratio is directly related to the type of cooling of the material. In rapid cooling the structure of the material is collapsed, and a high percentage of vitreous phase is obtained, which will make very reactive slag, and depending on the specific surface, it will have considerable pozzolanic properties [45]. Cooling may be considered rapid when the temperature drops from 1400 °C to 800 °C in a few minutes [46]. Water-cooled slags, such as the SS under study, are called granulated slags because the slag upon contact with water breaks down and forms grains similar to sand. This cooling is so fast that the atoms of the solid do not have time to orient themselves, and therefore have a high proportion of vitreous phase [47]. The proportion of vitreous phase that is usually present in these slags is between 85% and 95% after a grinding process gives them the appropriate specific surface. Cements with this type of material addition are so-called slag-cements [35]. According to EN 15167-1 [48], BFS must contain at least two-thirds of the vitreous phase slag and must have hydraulic properties when properly activated. In the so-called slag-cement collected in Reference [30], the activation of the slag is carried out directly by the Ca(OH)_2_, which is released in the process of hydration of the clinker by the alkalis produced and by the addition of gypsum to control the setting time. This type of hydration is produced in slag-cement when Ca(OH)_2_ is not released, AC3 is not formed, and the K_2_O and Na_2_O that are formed remain inside a crystalline network and not in a free state. Therefore, it can be said that slag-cements have special properties, especially in terms of the heat of hydration, compressive strength, and durability [33,45].

**Figure 11:**
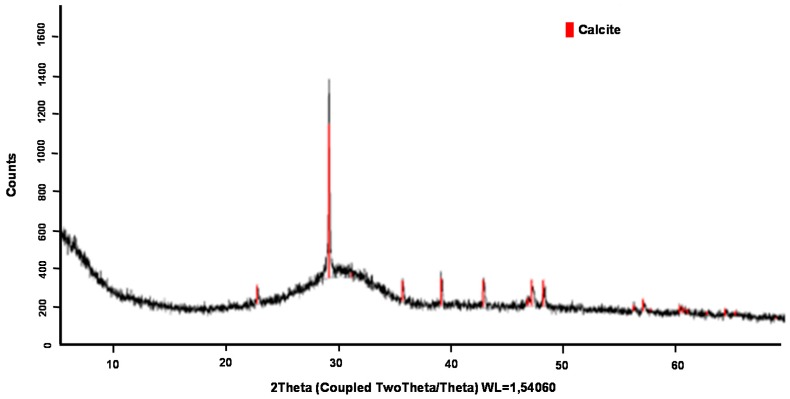
X-ray diffraction of BFS.

According to the X-ray diffraction tests, it can be said that BFS has a vitreous phase of 70% and a crystalline phase of 30%. The diffractogram corresponding to this material is shown in Figure 11 where the crystalline phases are represented as red-marked peaks (the crystalline peaks correspond to calcite). It should be noted that the BFS selected for the study did not have a very high percentage of vitreous phase compared to the maximum values of the BFS, which can usually reach values up to 90–95%. However, the proportions obtained were within the range collected in the standard and inside the range of values collected in the bibliography [29].

**Figure 12:**
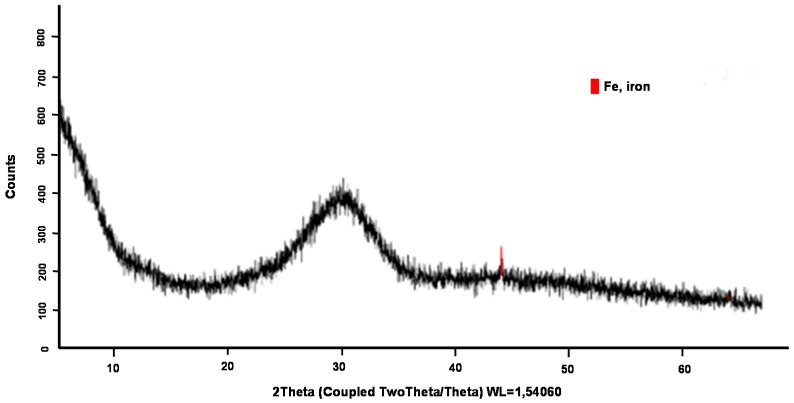
X-ray diffraction of SS.

For SS, the vitreous phase estimated by the X-ray tested was around 85–90%. Figure 12 shows the diffractogram obtained from the SS. Only a small crystalline peak marked in red in the image corresponding to crystalline iron was observed.

### 3.3. Mechanical, Physical, and Chemical Properties Results

In order to prove if the cement made from the addition of SS could be classified as blast furnace slag-cement according to Reference [29], the mechanical, physical, and chemical requirements included in this standard were evaluated.

#### 3.3.1. Mechanical Properties

**Figure 13:**
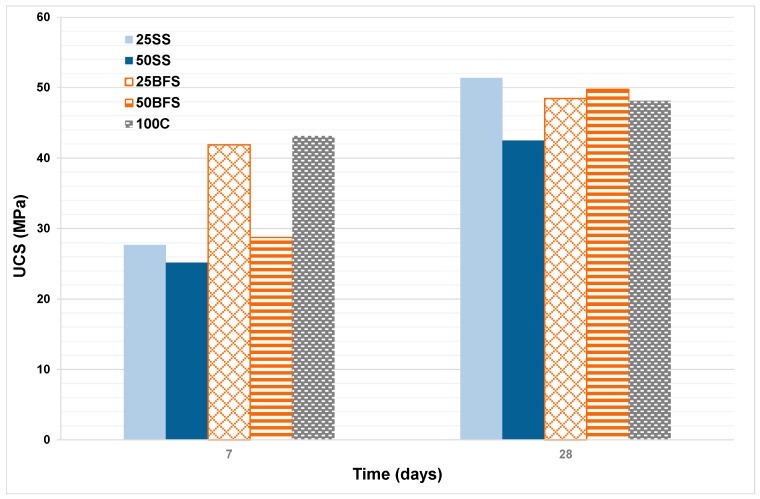
Evolution of the unconfined compressive strength (UCS) with time.

UCS is the most important property of mortars and concretes made from cement. The variation in the UCS at 7 and 28 days with the proportion of BFS and SS added in the compositions is shown in Figure 13; five samples were tested for each mix reference. At the early stages, the UCS of the samples with proportions of BFS and SS decreased, and were lower than in the cement composition alone. However, for the duration of 28 days, the UCS was higher in samples with addition than in the sample with only cement, at 25SS, which shows a higher UCS with values above 50 MPa. Usually, cement with the addition of slags has lower rates of hydration than common Portland cement, so that the speed and degree of hydration they can achieve will depend, among other factors, on the specific surface and on the particle size distribution. The specific surface area of the BFS used as addition to the cement was around 4000 and 6000 cm^2^/g. Particle sizes smaller than 10 microns have the fundamental function of contributing mechanical resistance in the early stages; whereas particles with sizes between 10 and 45 microns contribute to the resistance developed at more advanced stages [41,49]. All the phases of the cement which are capable of hydrating contribute to acquiring the structural properties [50]. A study on the hydration processes in cement concluded that the development of the initial resistance is controlled by the hydration of tricalcium silicate, (C3S) which is supported by tricalcium aluminate (C3A); while the dicalcium silicate (C2S) and the tetracalcic aluminoferrite (C4AF), which are phases with much slower hydration periods, make a significant contribution to the compressive strength at ages above 28 days. The hydration products that were formed when BFS was added to the Portland cement in its mixture with water were essentially the same as those produced in the hydration of Portland cement. These are basically hydrated calcium silicates. The slag hydration process was slower than the conventional Portland cement due to the vitreous character, causing the slag dissolution to be more difficult and require activation [46]. In cements with BFS additions, the first reaction was between cement and water, producing Ca(OH)_2_ of basic character, which serves as an activating element for slags. The slag hydration product is a C–S–H (calcium–silicate–water) gel with slightly less calcium and longer chains than that produced by the hydration of the cement. As a consequence of the slower hydration of the slags, the UCS at early ages of the slag-cements was not as strong as those of the conventional cements. However, at average ages of 28 days, the UCS was similar, achieving higher resistance values over a long term.

#### 3.3.2. Physical Properties

##### Setting Time

Two types of setting times were defined: the initial setting time corresponding to the period in which the stiffness of the paste starts, and the final setting time in which the paste loses its workability. During the initial setting time, the first component to react was the C3S, reducing the available water and the plasticity of the mixture due to the reaction of hydration. The C3A is more reactive than C3S, with its activity ceased by the gypsum, which is used as an addition to control the setting time to achieve the desired workability [44].

The initial and final setting times were characterized by the different proportions of the additions of SS (25SS and 50SS) to the cement, as well as the pastes obtained by the addition of BFS (25BFS and 50BFS) to the cement, and a 100% C, which serve as reference materials.

**Figure 14:**
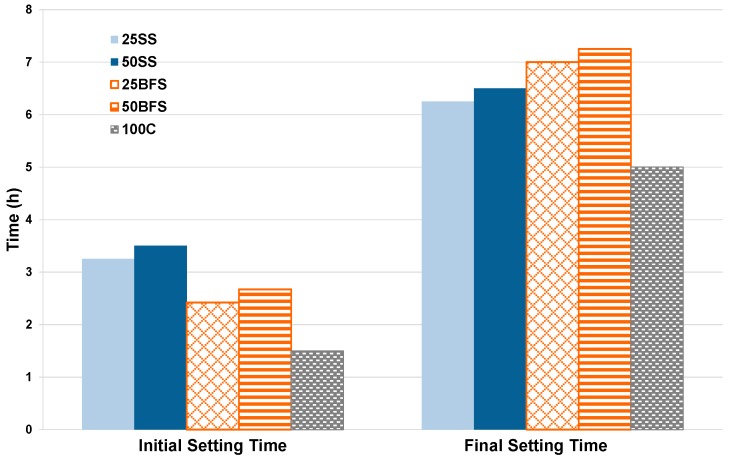
Evolution of the initial and final setting times.

Figure 14 shows the results obtained from the evolution of the setting time as a function of the ratio of BFS and SS; in this case three samples were tested for each one of the mix references. Note that the setting time increases with the increase in the proportion of addition to the C.

The initial setting time is limited by Reference [26] according to the classification made by the UCS results. The initial setting time must be greater than 75 min for cements classified with the compressive strength of 32.5. For cements of 42.5, this time should exceed 60 min, and in cases where the cement is classified as 52.5, the minimum allowed initial setting time should be above 45 min. As seen in the previous figure, the initial and final setting times of all compositions are within the limits required for each cement class.

##### Volume Stability

The volume stability affects the potential application of the material as a construction product since volume changes could cause structural failures. The volumetric expansion of the samples is limited to 10 mm according to European standard [30]. The samples tested were the same proportion of addition as the samples used for the setting time tests: 25SS, 50SS, 25BFS, and 50BFS. In no case was variation in the volume stable for the different samples. This may be due to the low content of MgO and free CaO present in the additions tested in the cement, which have a greater effect on the expansion of the mortars [44].

##### Heat Hydration

Heat of hydration is related to reactivity at early ages. The initial low values of UCS are indicative of a slower evolution of the heat of hydration. Therefore, the heat of hydration is a factor that has great importance at the practical level, since it has a self-accelerating effect on the hydration of the cement and can be better for use in cold environments. However, a high heat of hydration can have harmful effects on the internal part of the structure, especially in compact or solid concrete structures, which can cause mechanical stresses that may give rise to cracks in the structure. The heat of hydration, as well as other properties of the cement, is closely linked to its chemical composition, and above all, to the mineralogical composition of the four main components: C3S, C2S, C3A and C4AF. C3A has the highest heat of hydration of all of them, as well as the greatest variability, depending on the type of hydrate formed [44].

The composition of the mixtures tested and the results obtained are shown in Table 6.

According to the results obtained, it can be said that, in the case of the addition of BFS and SS, as the proportion of addition increases, the heat of hydration decreases. The decrease is more pronounced in the case of SS mixtures than in the case of mixtures with BFS. According to the general cement requirements, in order for a cement to be considered as having low hydration heat, the value of this parameter should not exceed the characteristic value of 270 J/g. The samples that could be considered as low-heat hydration cements were those in which the proportions of additions studied were above 50%.

#### 3.3.3. Chemical Properties

The results of chemical properties are shown in Table 7. All the samples were tested according to the requirements [29] and in all cases, the results obtained were below the required limit for slag-cements. In the case of calcination losses and the insoluble residue, the limit is 5.0% in both cases. For the sulfate content, the required limit is below 4.0% in slag-cements of all resistant categories, and the maximum chloride content allowed is 0.10%. According to the results obtained, it can be said that all the values were very far from the maximum allowed by the specific requirements of type III cements.

### 3.4. Emission of Dangerous Substances (Heavy Metals) and Emission of Radioactivity

Table 8 shows the values obtained in the leaching test, as well as the limit values that are considered acceptable by the standard.

As shown in Table 8, none of the elements measured in the leaching test exceeded the limit values included in the standard. In fact, none of the elements were close to the limit, and the use of these types of material in the building industry are not dangerous for human health or for the environment.

In the case of the radioactive test, according to recommendations [43], the limit of radiation allowed for a prolonged exposure of an individual is between 0.3 and 1.0 mSv/year. To comply with the regulations (based on the annual dose increment constraint of 0.3 mSv/year), the activity concentration index calculated for the product tested must comply with the criterion: I ≤ 1.0. The activity concentrations of radionuclides Ra-226, Th-232, and K-40 in samples of different proportion of SS, BFS, and cement are presented in Table 9.

The concentration results were higher in BFS than in SS. In any case, the activities of the different radionuclides were below the European recommendations for standard concrete and aerated concrete [43]. The results of Table 9 confirmed a potential for industrial use of SS because “I” was under this criterion.

## 4. Conclusions

The results obtained from this lab scale work have confirmed the technical feasibility of this innovative process to develop an innovative vitreous material from three industrial wastes such as CDW, MS, and AW. Indeed, a new waste mixture adjustment methodology was successfully defined in order to obtain an SS with similar chemical composition to BFSs. The result of this new adjustment proportioned a mix of 37 wt % CDW, 53 wt % MS, and 10 wt % AW. Regarding the mechanical properties studied, the samples tested after seven days showed worse UCS than the previous reported by BFS (25–28 MPa vs. 29–42 MPa). However, after 28 days this was reversed, and our proposed material showed stronger mechanical properties than BFS (43–52 MPa vs. 48–49 MPa). Both the physical and chemical properties, for example setting time and volume stability, were tested to confirm that the legislative requirement were successfully met. Additionally, environmental tests were performed to ensure the material viability obtained from waste, being not a future problem to be taken into account for the society.

Therefore, it can be concluded that the waste mixture of CDW, MS, and AW has potential recycling as a raw material in the manufacture of slag cements. With this, the foundations for future works are laid and further efforts will be focused on scaling-up our work to an industrial application in which valuable by-products could be obtained from waste in order to contribute to a circular economy policy.

## 5. Patents

Rodríguez-Galán, Mónica; Navarrete-Rubia, Benito; Vilches-Arenas, Luis Francisco; Leiva-Fernández, Carlos; Picón-bolaños, Juan Manuel; Díaz-Bautista, María Arantzazu. MATERIAL CEMENTANTE A PARTIR DE MEZCLAS DE RESIDUOS Y/O SUBPRODUCTOS INDUSTRIALES Y PROCEDIMIENTO DE FABRICACIÓN. 04.09.2017. Cementos Portland Valderrivas S.A. Cementos Portland Valderrivas (2592953B1).

## Figures and Tables

**Table 1:** Typical composition range of blast furnace slag (BFS).

**Chemical Composition**	SiO_2_	CaO	Al_2_O_3_	MgO	Fe_2_O_3_	SO_3_	Na_2_O	K_2_O
**Percentage (%)**	27–40	30–50	5–15	1–15	0.2–2.5	1–2.5	0.1–3	0.1–3

**Table 2:** Characteristics of the furnace.

**Maximum Operating Temperature**	**Continuous**	1650 °C
	**Intermittent**	1700 °C
**Indoor Chamber Capacity**		4.3 L
**Maximum Micro-Oven Temperature**		1500 °C
**Dimensions**	**Wide**	752 mm
	**Bottom**	360 mm
	**High**	800 mm
**Indoor Chamber Dimensions**	**Wide**	93.5 mm
	**High**	165 mm
**Useful Chamber for Samples**	**Diameter**	80 mm
	**High**	140 mm
**Electrical Characteristics**	**Voltage**	220 V
	**Amperage**	18 A
	**Power**	4 kW
	**Frequency**	50 Hz
	**Phases**	1

**Table 3:** Tested sampled proportions.

Mixture Proportion	Sample Name
25% SS + 75% C	25SS
50% SS + 50% C	50SS
25% BFS + 75% C	25BFS
50% BFS + 50% C	50BFS
100% C	100C

**Table 4:** Chemical composition of the wastes.

Chemical Composition (%)	CDW *	AW **	MS ***
Silicon dioxide (SiO_2_)	57.22	10.83	5.85
Aluminum oxide (Al_2_O_3_)	7.81	59.91	0.39
Ferric oxide (Fe_2_O_3_)	3.45	2.10	0.27
Calcium oxide (CaO)	12.90	2.26	48.92
Manganese oxide (MnO)	0.06	0.24	0.01
Magnesium oxide (MgO)	1.50	4.82	0.16
Sodium oxide (Na_2_O)	1.02	2.69	0.80
Potassim oxide (K_2_O)	1.51	1.10	0.14
Titanium oxide (TiO_2_)	0.46	0.84	N.D.
Phosphorous pentoxide (P_2_O_5_)	0.10	0.04	0.07
Sulfur trioxide (SO_3_)	1.65	0.11	0.17
Loss on ignition (L.O.I)	10.82	12.66	43.09

* CDW: construction and demolition waste; ** AW: aluminium waste; *** MS: Mussel shell.

**Table 5:** Chemical composition of SS and BFS.

Chemical Composition (%)	SS	BFS
Silicon dioxide (SiO_2_)	38.05	33.67
Aluminum oxide (Al_2_O_3_)	11.42	9.95
Ferric oxide (Fe_2_O_3_)	1.77	0.30
Calcium oxide (CaO)	42.63	42.93
Manganese oxide (MnO)	0.07	0.25
Magnesium oxide (MgO)	1.44	6.90
Sodium oxide (Na_2_O)	1.36	0.23
Potassim oxide (K_2_O)	0.79	0.40
Titanium oxide (TiO_2_)	0.32	0.48
Phosphorous pentoxide (P_2_O_5_)	0.10	-
Sulfur trioxide (SO_3_)	0.68	0.44
L.O.I	0.21	2.75

**Table 6:** Heat hydration values.

Sample	Heat Hydration (J/g)
25SS	315 ± 5
50SS	255 ± 5
25BFS	295 ± 5
50BFS	265 ± 5

**Table 7:** Chemical properties.

**Sample**	25SS	50SS	25BFS	50BFS
**Loss on Ignition (%)**	1.99	1.19	1.72	1.09
**Insoluble Residue (%)**	0.29	0.23	0.28	0.24
**Sulfate Ion (%)**	2.1	1.4	2.3	1.7
**Chloride Ion (%)**	<0.1	<0.1	<0.1	<0.1

**Table 8:** Values of leaching test obtained according to the NEN 7345 (mg/m^2^).

Chemical Compound	25SS	50SS	25BFS	50BFS	100C	Limit Value
Sb	1.34	1.48	1.41	1.31	1.37	8.7
As	0.49	0.54	0.51	0.48	0.50	260
Cd	0.12	0.13	0.13	0.12	0.12	3.8
Cr	3.86	4.5	2.04	1.55	1.92	120
Co	0.12	0.13	0.13	0.12	0.12	60
Cu	0.12	0.13	0.13	0.12	0.12	98
Hg	0.24	0.27	0.26	0.24	0.25	1.4
Mo	0.12	0.13	0.13	0.12	0.12	144
Ni	0.37	0.40	0.38	0.36	0.37	81
Se	0.49	0.54	0.51	0.48	0.50	4.8
Sn	0.49	0.54	0.51	0.48	0.50	50
V	5.51	5.42	3.04	3.17	2.72	320
Zn	0.12	0.13	0.13	0.12	0.12	800
Ba	2.49	4.94	12.76	6.76	7.56	1,500
Pb	0.85	0.94	0.9	0.84	0.87	400
Co	0.12	0.13	0.13	0.12	0.12	60

**Table 9:** Activity concentrations (Bq/kg) of radionuclides Ra-226, Th-232, K-40 and the activity concentration index, I, of SS, BFS, and cement.

Activity Concentrations (Bq/kg)	25SS	50SS	25BFS	50BFS	100C
K-40	80	94	57	55	54
Ra-226	5.6	6.9	19.8	39	5.4
Th-232	5.8	9.1	8.8	14	5.5
Activity Concentration Index (I)	0.074	0.100	0.129	0.218	0.064

## References

[1] Ke X., Criado M., Provis J.L., Bernal S.A. (2018). Slag-based cements that resist damage induced by carbon dioxide. ACS Sustain. Chem. Eng..

[2] Lee H., Hanif A., Usman M., Sim J., Oh H. (2018). Performance evaluation of concrete incorporating glass powder and glass sludge wates as supplementary cementing material. J. Clean. Prod..

[3] Wang Y., Shao Y., Matovic M.D., Whalen J.K. (2016). Recycling combustion ash for sustainable cement production: A critical review with data-mining and time-series predictive models. Constr. Build. Mater..

[4] Biskri Y., Achoura D., Chelghoum N., Mouret M. (2017). Mechanical and durability characteristics of high performance Concrete containing steel slag and crystalized slag as aggregates. Constr. Build. Mater..

[5] Jani Y., Hogland W. (2014). Waste glass in the production of cement and concrete—A review. J. Environ. Chem. Eng..

[6] Paya J., Monzó J., Borrachero M.V., Velázquez S. (2013). Cement equivalence factor evaluations for fluid catalytic cracking catalyst residue. Cem. Concr. Comp..

[7] Rosales J., Cabrera M., Agrela F. (2017). Effect of stainless steel slag waste as a replacement for cement in mortars. Mechanical and statistical study. Constr. Build. Mater..

[8] Ding B., Wang H., Zhu X., He X.Y., Tan Y., Liao Q. (2017). Phase change cooling and crystallization characteristics of blast furnace slags with various MgO/Al_2_O_3_ ratios. ACS Energy Fuels.

[9] Vlcek J., Tomkova V., Ovcacikova H., Ovcacik F., Topinkova M., Matejka V. (2013). Slags from Steel Production: Properties and Their Utilization. Metalurgija.

[10] Liu J., Yu Q., Zuo Z., Yang F., Duan W., Qin Q. (2017). Blast furnace slag obtained from dry granulation method as a component in slag cement. Constr. Build. Mater..

[11] Erdogan S.T., Kocak T.C. (2017). Influence of slag fineness on the strength and heat evolution of multiple-clinker blended cements. Constr. Build. Mater..

[12] Lin K.L., Wang K.S., Tzeng B.Y., Lin C.Y. (2004). The hydration characteristics and utilization of slag obtained by the vitrification of MSWI fly ash. Waste Manag..

[13] Lin K.L., Wang K.S., Lin C.Y., Lin C.H. (2004). The hydration properties of pastes containing municipal solid waste incinerator fly ash slag. J. Hazard. Mater..

[14] Lin K.L., Lin D.F., Wang W.J., Chang C.C., Lee T.C. (2014). Pozzolanic reaction of a mortar made with cement and slag vitrified from MSWI ash-mix and LED sludge. Constr. Build. Mater..

[15] Lee T.C., Wang W.J., Shih P.Y. (2008). Slag-cement mortar made with cement and slag vitrified from MSWI fly-ash/scrubber-ash and glass frit. Constr. Build. Mater..

[16] Lee T.C., Rao M.K. (2009). Recycling municipal incinerator fly—And scrubber—Ash fused slag for the substantial replacement of cement in cement—Mortars. Waste Manag..

[17] Lee T.C. (2009). Recycling of municipal incinerator fly-ash slag and semiconductor waste sludge as admixtures in cement mortar. Constr. Build. Mater..

[18] Lee T.C., Li Z.S. (2010). Conditioned MSWI ash-slag-mix as a replacement for cement in cement mortar. Constr. Build. Mater..

[19] Bhardwaj B., Kumar P. (2017). Waste foundry sand in concrete: A review. Constr. Build. Mater..

[20] Bahoria B.V., Parbat D.K., Naganaik P.B. (2013). Replacement of natural sand in concrete by waste products: A State of Art. J. Environ. Res. Dev..

[21] Actualización del Catálogo de Residuos Utilizables en Construcción. http://www.cepco.es/Uploads/docs/Actualizacion%20del%20catalogo%20de%20residuos%20utilizables%20en%20construccion.pdf.

[22] Medina C., Banfill P.F.G., Sánchez de Rojas M.I., Frías M. (2013). Rheological and calorimetric behaviour of cement blended with containing ceramic sanitary ware and construction/demolition waste. Constr. Build. Mater..

[23] Tayibi H., Pérez C., López F.A., López-Delgado A. (2005). Evolution of mechanical properties of a residue from the secondary aluminum remelting industry stabilized with gypsum. Rev. Metal..

[24] Carnero López M., Fernández Rodríguez M.E., Carreira Pérez X.C., Méndez Lodos M. Mezclas de Zahorras naturales y concha de mejillón para firmes forestales. Proceedings of the XIII Congreso Internacional de Ingeniería de Proyectos.

[25] Kuo W.T., Wang H.Y., Shu C.Y., Su D.S. (2013). Engineering properties of controlled low-strength materials containing waste ouster shells. Constr. Build. Mater..

[26] Wang H.Y., Kuo W.T., Lin C.C., Po-yo C. (2013). Study of the material properties of fly ash added to oyster cement mortar. Constr. Build. Mater..

[27] Leiva Fernández C., Vale Parapar J., Muñoz Gil F., Fernández Pereira C., Vilches Arenas L.F. (2009). Obtención de Materiales Resistentes al Fuego a Partir de Residuos Procedentes de la Industria. Conservera. Patent.

[28] Li G., Xu X., Chen E., Fan J., Xiong G. (2014). Properties of cement-based bricks with oyster-shells ash. J. Clean. Prod..

[29] (2005). EN (European Standard) 197-4:2005. Cement. Part 4: Composition, Specifications and Conformity Criteria for Low Early Strength Blastfurnace Cements.

[30] (2011). EN (European Standard) 197-1:2011. Cement. Part 1: Composition, Specifications and Conformity Criteria for Common Cements.

[31] Tovar Rodríguez G. (2011). Estudio de Morteros de Árido Reciclado fino en Matrices con Cemento Portland y en Matrices de Escoria de alto Horno Activada Alcalinamente. Master’s Thesis.

[32] Moranville-Regourd M., Lea’s Chemistry of Cement and Concrete (1998). Cements Made from Blast Furnace Slag.

[33] Calleja J. (1982). Escorias y cementos siderúrgicos. Mater. Constr..

[34] Zhu J., Zhong Q., Chen G., Li D. (2012). Effect of particlesize of blast furnace slag on properties of Portland cement. Procedia Eng..

[35] (1993). Escorias de alto horno. Composición y comportamiento Hidráulico. Mater. Constr..

[36] (2010). EN (European Standard) 196-6:2010 Methods of Testing Cement. Part 6: Determination of Fineness.

[37] (2005). EN (European Standard) 196-1: 2005 Methods of Testing Cement. Part 1: Determination of Strength.

[38] (2005). EN (European Standard) 196-3:2005 + A1: 2008 Methods of Testing Cement. Part 3: Determination of Setting Time and Soundness.

[39] (2003). EN (European Standard) 196-8: 2003 Methods of Testing Cement. Part 8: Heat of Hydration. Solution Method.

[40] (2005). EN (European Standard) 196-2: 2005 Methods of Testing Cement. Part 2: Chemical Analysis of Cement.

[41] (2010). EN (European Standard) 80123:2010 Methods of Cement Testing. Potentiometric Determination of Chlorides.

[42] (2005). NEN 7345 (1995). Leaching Characteristics of Solid Earthy and Stony Building and Waste Materials. Leaching Test. Determination of the Leaching of Inorganic Components from Building and Monolithic Waste Materials with the Diffusion.

[43] European Commission Radiation Protection (1999). Radiological Protection Principles Concerning the Natural Radioactivity of Building Materials, in 112.

[44] Blanco Álvarez F. (2011). Tecnología de Cementos, Vidrio y Cerámicas. Escuela Superior de Ingenieros de Minas.

[45] Elías Castells X. (2005). Tratamiento y Valorización Energética de Residuos.

[46] Reino García H. Supercem. Experiencia de Holcim (España) con cementos con escorias de alto horno altamente adicionados. Proceedings of the Ciclo de Conferencias. Patología de cimentaciones, estructuras y hormigones, IETCC-CSIC, E.T.S. Arquitectura de Valencia.

[47] (2010). Actualización del Catálogo de Residuos 2010 (Para Varadero). Centro de Estudios y Experimentación de Obras Públicas (CEDEX). Ministerio de Medio Ambiente, Medio Rural y Marino. Ministerio de Fomento. Spain. Ficha Técnica. Escorias de horno alto. HOLCIM. Diciembre. http://www.cedex.es/NR/rdonlyres/F4AC0E92-C7BF-44FE-A972-0CB07F24AFAB/119857/ESCORIASDEACERIALD.pdf.

[48] (2006). EN 15167:2006 Ground Granulated Blast Furnace Slag for Use in Concrete, Mortar and Grout. Part 1: Definitions, Specifications and Conformity Criteria.

[49] Castellano C.C., Bonaretti V.L., Irassar E.F. (2013). Cemento Mezclas: Influencia del tamaño de las partículas de escoria. Concreto y Cemento. Investig. Desarro..

[50] Taylor H.F.W. (1997). Cement Chemistry.

